# Comparative Proteome Analysis of Epicardial and Subcutaneous Adipose Tissues from Patients with or without Coronary Artery Disease

**DOI:** 10.1155/2019/6976712

**Published:** 2019-08-25

**Authors:** Yu xing Zhao, Hui juan Zhu, Hui Pan, Xue mei Liu, Lin jie Wang, Hong bo Yang, Nai shi Li, Feng ying Gong, Wei Sun, Yong Zeng

**Affiliations:** ^1^Key Laboratory of Endocrinology of National Health Commission, Department of Endocrinology, Peking Union Medical College Hospital, Chinese Academy of Medical Sciences, Beijing, China; ^2^Institute of Basic Medical Sciences, Chinese Academy of Medical Sciences, School of Basic Medicine, Peking Union Medical College, Beijing, China; ^3^Department of Cardiology, Beijing Anzhen Hospital, Capital Medical University, Beijing, China

## Abstract

**Background and Aims:**

Owing to its unique anatomical structure and metabolism, epicardial adipose tissue (EAT) has attracted amount of attention in coronary artery disease (CAD) research. Here, we analyzed differences in proteome composition in epicardial (EAT) and subcutaneous adipose tissues (SAT) from patients with or without CAD.

**Methods:**

EAT and SAT samples were collected from 6 CAD patients and 6 non-CAD patients. Isobaric Tagging for Relative and Absolute Quantitation (iTRAQ) analysis combined with liquid chromatography tandem-mass spectrometry (LC-MS/MS) was performed to identify the differentially expressed proteins.

**Results:**

In total, 2348 proteins expressed in EAT and 2347 proteins expressed in SAT were separately identified. 385 differentially expressed proteins were found in EAT and 210 proteins were found in SAT in CAD patients compared to non-CAD patients. Many proteins differentially expressed in EAT of CAD patients were involved in biological functions associated with CAD development such as cell-to-cell signaling and interaction, inflammatory response, and lipid metabolism. Differential expressions of proteins (MMP9, S100A9, and clusterin) in EAT or SAT were involved in several signaling pathways such as mitochondrial dysfunction, acute phase inflammation, and LXR/RXR activation, which was confirmed by western blotting, and similar results were obtained.

**Conclusions:**

The largest profiles of differentially expressed proteins in EAT and SAT between CAD patients and non-CAD patients were identified. The significant signal pathways, mitochondrial dysfunction, and LXR/RXR activation, which differential proteins were involved in, were firstly found to play roles in EAT of CAD patients, and clusterin was firstly found to be upregulated in EAT of CAD patients.

## 1. Introduction

Coronary artery disease (CAD) is a severe chronic disease, which threatens human health. Adipose tissue has been reported to play an important role in the development and progression of atherosclerosis, and it is involved in regulating the inflammatory response and energy metabolism by secreting a variety of adipokines such as adiponectin, leptin, and resistin [1–3].

Adipose tissue is usually divided into visceral adipose tissue (VAT) and subcutaneous adipose tissue (SAT) according to its location. It has been widely accepted that VAT presents an adverse metabolic risk profile to the development of cardiovascular diseases and metabolic disorders such as diabetes and metabolic syndrome [4, 5]. In contrast, SAT mainly acts to resist the inflammatory response and protect the body from the metabolic disorders [6]. Epicardial adipose tissue (EAT) is such a kind of unusual visceral fat depot which locates between the myocardium and visceral pericardium. Increasement in epicardial fat volume was associated with greater coronary artery calcification progression, suggesting that epicardial fat might be a CAD risk predictor [7, 8].

Although EAT and SAT exert different roles in the metabolic disorders, only a few studies have investigated the differences in their proteomes. Salgado-Somoza et al. [9] identified 5 differentially expressed proteins in EAT compared to SAT in Spanish patients with cardiovascular disease by means of two-dimensional (2D) gel electrophoresis and MALDI-TOF/TOF (Matrix-Assisted Laser Desorption/Ionization Time of Flight) spectroscopy. They demonstrated that EAT suffered greater oxidative stress than SAT in patients with cardiovascular diseases and that apolipoprotein A-I and glutathione S-transferase P expression levels in EAT were higher than those in SAT in Spanish patients with cardiovascular diseases through one- or two-dimensional electrophoresis and mass spectrometry [10]. However, the numbers of differentially expressed proteins identified from EAT and SAT were small owing to limitations of susceptibility and stability in methodology. It is limited to have deeper recognition of EAT based on past research. Since the development of Isobaric Tagging for Relative and Absolute Quantitation (iTRAQ) coupled with liquid chromatography tandem-mass spectrometry (LC-MS/MS) as a high-resolution and high-throughput mass spectrometry technique, it became widely used to identify and quantify proteomes of different tissues or cell type. Therefore, in the present study, the differentially expressed proteomes in EAT and SAT from patients with and without CAD were investigated by means of iTRAQ and LC-MS/MS.

## 2. Materials and Methods

### 2.1. Patients

In total, 12 patients including 6 patients with CAD (M/F = 4/2; age, 59.83±7.57) and 6 patients without CAD (M/F = 2/4; age, 49.6±8.38) were recruited from Peking Union Medical College Hospital (PUMCH). CAD patients underwent coronary angiography, which showed coronary stenosis ≥ 70% to confirm the diagnosis, and underwent coronary artery bypass grafting surgery. Six patients without CAD showed no stenosis by the coronary angiography and underwent valve surgery due to their heart valve disease.

Exclusion criteria for the above patients were as follows: severe infection, malignant tumors, autoimmune disease, diabetes mellitus, dissection of the aorta, aneurysm, severe dysfunction of liver or kidney, and use of glucocorticoids, immunosuppressive agents, or chemotherapy drugs. Study protocol conforms to the ethical guidelines of the 1975 Declaration of Helsinki and was approved by the Ethics Committee of PUMCH, China (S-K205). All participants provided written informed consent before entering the project.

### 2.2. Samples

EAT samples (150–200 mg wet weight) were obtained from the upper region between the myocardium and visceral pericardium of the right ventricle, and SAT samples (200–300 mg wet weight) were harvested from the median sternotomy site during surgery. Adipose tissue samples were washed by precooled physiological saline, and blood vessels were removed before freezing the samples in liquid nitrogen.

### 2.3. Extraction of Protein

Proteins were extracted from adipose tissue samples (100 mg) with the protein extraction kit (Applygen Technologies Inc, Beijing, China) following the manufacturer's protocol and dissolved in 100 *μ*l lysate containing 1% Tris (pH 6.8), 42% urea, 15.4% thiourea, and 1% dithioerythritol (Sigma-Aldrich, St. Louis, MO, USA). Bradford method was used to determine the protein concentration.

### 2.4. Protein Digestion and iTRAQ-Labeling

Proteins (100 *μ*g) from each adipose tissue sample were weighted and assigned to an EAT-CAD group (EAT from CAD patients), a SAT-CAD group (SAT from CAD patients), an EAT-non-CAD group (EAT from non-CAD patients), and a SAT-non-CAD group (SAT from non-CAD patients). The protein samples were digested with filter-aided sample preparation method [11]. Briefly, the protein samples were first reduced with 20mM DTT at 95°C for 3-5 min and then washed once with 8M urea on 10KDa filter (Pall) at 14,000 g for 40 min. The mixture was alkylated with 55 mM iodoacetamide for 30 min in the dark, washed twice with 8M urea. The protein samples were washed with 50 mM ammonium bicarbonate once and then digested with trypsin (1 *μ*g/50 *μ*g protein) overnight at 37°C. The peptide mixture was desalted on OASIS C18 solid phase extraction column (Waters). All samples were lyophilized for further MS analysis. The digested samples were labeled by iTRAQ. Labeling was performed according to the manufacturer's protocol (ABsciex). The labeled samples were mixed into one sample at the same amount and lyophilized. The iTRAQ labeled mixed samples were, respectively, redissolved in buffer A (pH 11, 5 mM Citric acid adjusted by NH4OH). The sample was analyzed by RP C18 LC column from waters (3.0mm×150mm, 3*μ*m). The eluted gradient was 5-30% buffer B (pH 11, 90% ACN; flow rate 1 mL/min, 5 mM Citric acid adjusted by NH4OH) for 60 min. A fraction was collected every minute; thus total 60 fractions were collected. To reduce the MS analysis time, every three fractions were pooled and finally 20 samples were lyophilized for MS analysis.

### 2.5. LC-MS/MS

The lyophilized samples were, respectively, redissolved in 0.1% formic acid (buffer A) before MS analysis. The samples were analyzed by RP C18 capillary LC column from Michrom Bioresources (100*μ*m×150mm, 3*μ*m). The eluted gradient was 5–30% buffer B (0.1% formic acid, 99.9% ACN; flow rate, 0.5 *μ*L/min) for 100 min.

MS Data was acquired in TripleTOF 5600 MS using an ion spray voltage of 3 kV, curtain gas of 20 PSI, nebulizer gas of 30 PSI, and an interface heater temperature of 150°C. The precursors were acquired in 500 ms ranging from 350 to 1250 Da, and the product ion scans were acquired in 50 ms and ranged from 100 to 1800 Da. A rolling collision energy setting was used. Total 30 product ion scans were collected if exceeding a threshold of 125 counts per second (counts/s) and with a +2 to +5 charge-state for each cycle.

### 2.6. Data Processing

The tandem mass spectral peak lists were converted to the Mascot generic format by Mascot software version 2.3.02 (Matrix Science, London, UK). The data sets were searched individually and in combination by Mascot v2.3.02 against the Swiss-Prot human database from UniProt (http://www.ebi.ac.uk/swissprot/) [12]. Carbamidomethylation (C) and iTRAQ 4-plex label was set as a fixed modification. The following constraints were used in the study: two missed tryptic cleavages were allowed; a peptide and production tolerance of 0.05 Da were used for searches; 1% false positive rate at protein level and each protein with 2 unique peptides were applied. The peptide abundances were normalized in different reporter ion channels of MS/MS scan. The protein abundance ratio was based on unique peptide results [13, 14].

Comparative proteomic analysis of adipose tissue proteins was performed on EAT and SAT from CAD and non-CAD groups. For identification of differentially expressed proteins, cutoffs for the fold change and P value were set to 1.5 and 0.05, respectively (fold change is the ratio of intensity of protein expression in CAD adipose tissue to that in non-CAD adipose tissue), which were considered the thresholds to minimize biological and technical errors.

### 2.7. IPA Network Analysis

The IPI accession numbers of differential proteins were inserted into the Ingenuity Pathway Analysis (IPA) software (Ingenuity Systems, Mountain View, CA), which categorizes gene products based on the location of the protein within cellular components. Possible biochemical, biological, and molecular functions could be suggested by IPA [14, 15].

### 2.8. Western Blotting

Proteins (30 *μ*g) from each sample were subjected to SDS-PAGE (Sodium Dodecyl Sulfate Polyacrylamide Gel Electrophoresis) (10% acrylamide, w/v) and transferred to PVDF (polyvinylidene fluoride) membranes (Amersham Biosciences, Uppsala, Sweden). Nonspecific binding sites were blocked with 5% (v/v) nonfat milk in 50 mM Tris-HCl, pH 7.4, containing 150 mM NaCl, 0.1% (w/v) Triton X-100 at 20°C at room temperature for 1 h. Immunological detection was performed using the anti-MMP9 antibody at 1:1000 dilution (Cell Signaling Technology, Inc, Danvers, MA, USA), anti-S100A9 at 1:250 dilution (HPA004193, Sigma-Aldrich), and anti-clusterin at 1:100 dilution (Santa Cruz Biotechnology, Santa Cruz, CA, USA). After washing three times, blots were further incubated with a horseradish peroxidase-conjugated secondary antibody (1: 5000 dilution) at room temperature for 1 h. Protein intensities were determined using densitometry (LabWorks, UVP, Inc., Upland, CA, USA). Differences with p values<0.05 were considered to be statistically significant.

### 2.9. Statistical Analysis

Clinical data and the result of western blot were analyzed by IBM SPSS Statistics 19 software (Armonk, New York, USA). The clinical data from two groups correspond to normal distribution using Kolmogorov-Smirnov test and normal Q-Q Plot. Comparisons between groups were performed by independent-samples T test where differences with* p *< 0.05 were considered statistically significant for clinical data. Differential protein data was input into IPA (Ingenuity Pathway Analysis Software, Mountain View, CA) and analyzed.

### 2.10. Workflow of the Differential Proteomic Analysis

**Figure 1:**
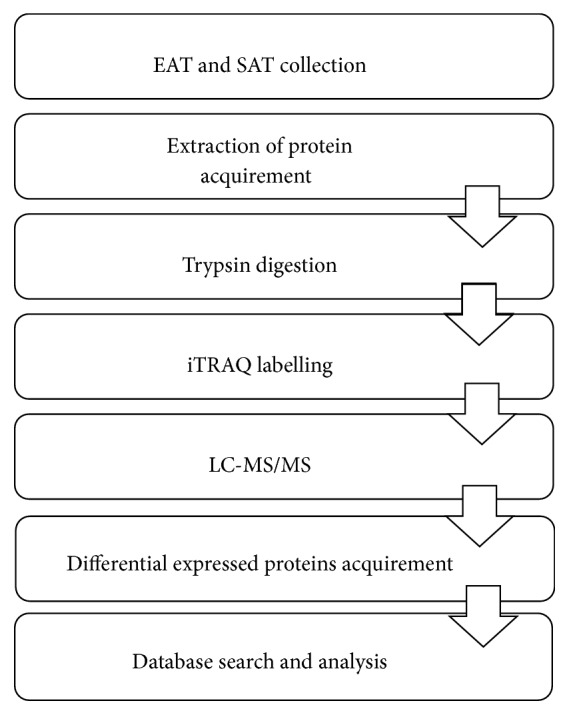
*Workflow of differential proteomic analysis of EAT and SAT samples from CAD and non-CAD patient populations*. Epicardial adipose tissue (EAT) and subcutaneous adipose tissue (SAT) samples were collected from 6 CAD patients and 6 non-CAD patients. Total proteins of adipose tissue were extracted and quantified via iTRAQ-coupled 2D LC-MS/MS. The differentially expressed proteins between the CAD and non-CAD groups were identified and analyzed by MASCOT and IPA (Ingenuity Pathway Analysis Software). Western blot was used to verify the selected differentially expressed proteins.

Workflow of the differential proteomic analysis of EAT and SAT in the CAD and non-CAD groups is displayed in Figure 1.

## 3. Results

### 3.1. Characteristics of the Study Population

The baseline characteristics and biochemical values for 12 patients are shown in Table 1. Six patients from the CAD group and one from the non-CAD group were also diagnosed with hypertension. None of patients in either group was diagnosed with diabetes. There were no significant differences in age, BMI, waist circumference, fasting blood glucose, and lipid profiles between the CAD and non-CAD groups as expected. Statin was used by three patients in the CAD group.

### 3.2. Differential Proteomic Analysis of EAT and SAT in the CAD and Non-CAD Groups

In total, 2348 proteins expressed in EAT and 2347 proteins expressed in SAT were separately identified, and 1949 proteins were identified in both EAT and SAT. Comparative proteomic analysis of adipose tissue proteins was, respectively, performed on EAT (EAT-CAD vs EAT-non-CAD) and SAT (SAT-CAD vs SAT-non-CAD).

#### 3.2.1. Proteomic Analysis of Differentially Expressed Proteins from EAT

**Figure 2:**
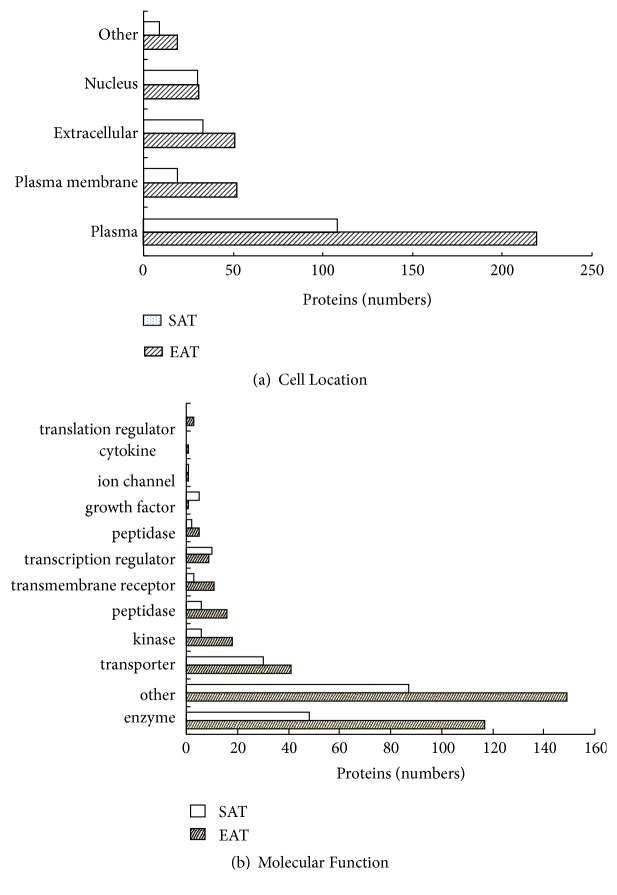
*Classification of differentially expressed proteins*. Differentially expressed proteins between CAD and non-CAD group in EAT and SAT were analyzed by Mascot. (a) Cell component of differential proteins from EAT and SAT. (b) Molecular function of differentially expressed proteins in EAT (epicardial adipose tissue) and SAT (subcutaneous adipose tissue).

385 differentially expressed proteins in EAT were found in the CAD group compared to the non-CAD group, which included 214 upregulated proteins and 171 downregulated proteins, while 13 proteins were unknown through Mascot software. Further analysis focusing on cellular localization of identified proteins showed that a high proportion of the proteins were annotated to cytoplasm (n = 219) as displayed in Figure 2(a). Based on protein function, these differentially expressed proteins were classified into 12 groups, of which enzymes formed the largest group (n = 117) as shown in Figure 2(b).

**Figure 3:**
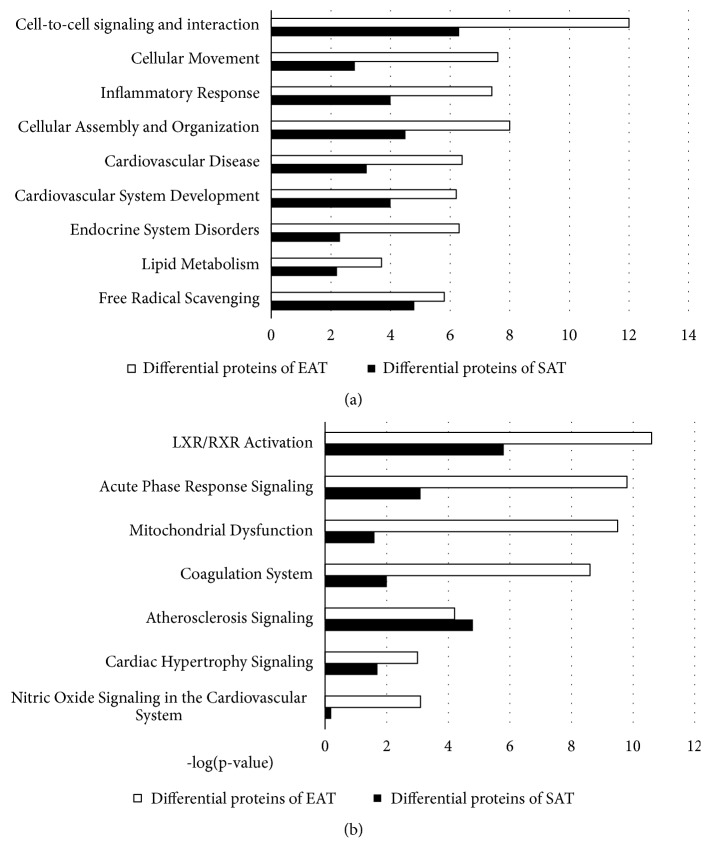
*The amounts of differentially expressed proteins involved in significant biological functions and signal pathways of EAT and SAT between the CAD and non-CAD groups*. Ingenuity Pathway Analysis was performed to analyze the function and signal pathway of differentially expressed proteins. (a) Differentially expressed proteins in EAT and SAT involved in several significant biological functions. (b) Differentially expressed proteins in EAT and SAT involved in several significant signal pathways.

To gain a better understanding of 372 differentially expressed proteins from EAT, a detailed analysis of their biological functions and involvement in signaling pathways was performed using IPA software. The results demonstrated that the main biological functions of these proteins included cell-to-cell signaling and interaction, cellular movement, and Cellular Assembly and Organization as shown in Figure 3(a), while the main signaling pathways included LXR/RXR activation, acute phase response, and mitochondrial dysfunction as presented in Figure 3(b).

#### 3.2.2. Proteomic Analysis of Differentially Expressed Proteins from SAT

In total, 210 differentially expressed proteins in SAT were found in the CAD group compared to the non-CAD group, including 71 upregulated proteins and 139 downregulated proteins, while 11 proteins were unknown through Mascot software. Cellular localization of these proteins is presented in Figure 2(a). Based on function of proteins, differential proteins from SAT were classified as enzymes (n = 48), transporters (n = 30), and transcriptional regulators (n = 10), among others as shown in Figure 2(b).

Similarly, detailed analyses of biological function and signaling pathways, in which differentially expressed proteins from SAT were involved, were performed using IPA software. The results demonstrated that the main biological functions of the differentially expressed proteins were cell-to-cell signaling and interaction, free radical scavenging, and cellular assembly and organization as shown in Figure 3(a), and the main signaling pathways in which the differentially expressed proteins were involved were LXR/RXR activation, atherosclerosis signaling, and acute phase response as presented in Figure 3(b).

#### 3.2.3. Differentially Expressed Proteins from EAT and SAT Have Been Involved in Biological Functions and Metabolic Signaling Pathways Related to Coronary Artery Atherosclerosis

Coronary artery disease-related biological functions were significant for exploring the mechanism of coronary artery atherosclerosis which attracted our more attention and were summarized including cell-to-cell signaling and interaction, inflammatory response, metabolic disorder, inflammatory response, lipid metabolism, and free radical scavenging as shown in Table 2. 36 differentially expressed proteins from EAT (such as A2M, C3) and 9 differentially expressed proteins from SAT (such as BPI) were involved in cell-to-cell signaling and interactions including cell adhesion, adhesion of inflammatory cells, phagocytosis, etc. 18 differentially expressed proteins from EAT (such as ACOT1) and 4 differentially expressed proteins from SAT (such as ALOX5) participated in lipid metabolism, while 22 differentially expressed proteins from EAT and 10 differentially expressed proteins from SAT were involved in inflammatory response. Obviously, there were more differentially expressed proteins in EAT than in SAT that were involved in these biological functions.

Coronary artery atherosclerosis-related signaling pathways which differentially expressed proteins were involved in included LXR/RXR activation, acute response signaling, and mitochondrial dysfunction. As shown in Table 2, there were 19 differentially expressed proteins from EAT and 10 differentially expressed proteins from SAT involved in LXR/RXR activation, 21 differentially expressed proteins from EAT and 8 differentially expressed proteins from SAT involved in acute phase response signaling, and 19 differentially expressed proteins from EAT and 5 differentially expressed proteins from SAT involved in mitochondrial dysfunction. Most of differentially expressed proteins in EAT from CAD patients were upregulated in LXR/RXR activation and acute response signaling, while expressions of differential proteins of SAT from CAD patients were downregulated. In the mitochondrial dysfunction signaling pathway, most of proteins differentially expressed in EAT and some differentially expressed proteins in SAT from CAD patients were downregulated as shown in Table 2.

### 3.3. Validation of Proteomic Data by Western Blot

**Figure 4:**
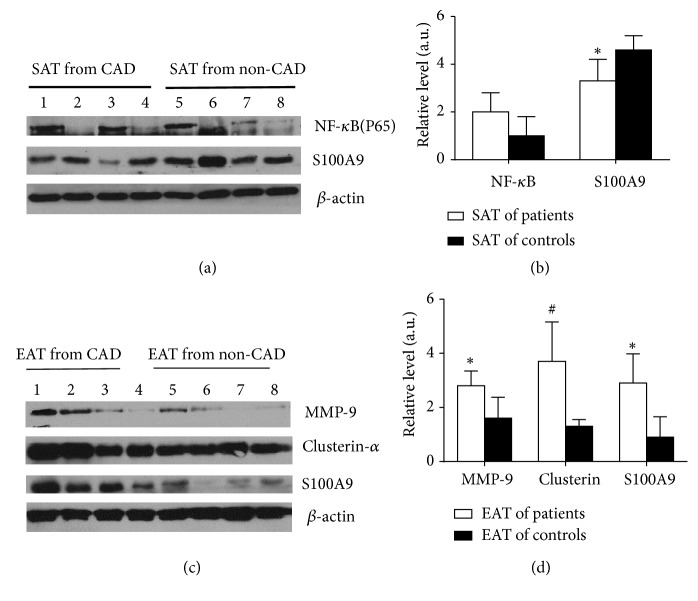
*MMP-9, clusterin, and S100A9 expression in EAT and S100A9 and NF-κB p65 expression in SAT from CAD and non-CAD groups as measured by western blot*. Western blot analysis compared MMP-9, clusterin, and S100A9 in EAT and S100A9 and NF-*κ*B p65 in SAT from CAD and non-CAD groups. (a) and (b), gel images and relative level of protein expression in SAT between CAD and non-CAD groups. (c) and (d), gel images and relative level of protein expression in EAT between CAD and non-CAD groups. *β*-actin was used as a loading control. The blot was repeated three times with n = 4 for each group. (b) Densitometric analysis of the blot in (a). *∗*P<0.05 vs non-CADs group; #P<0.01 vs non-CADs group. a.u.: arbitrary unit.

Comprehensive functional analyses of differentially expressed proteins, which were involved in inflammatory response, lipid metabolism, and LXR/RXR signal pathway, were made. To further confirm our observations, western blotting analysis was performed for four important proteins, including matrix metalloproteinase 9 (MMP9), clusterin, and migration inhibitory factor-related protein 9 (S100A9/MRP-9) in EAT and S100A9 and nuclear factor-kappa B p65 (NF-*κ*B p65, RELA) in SAT. MMP9 as an important inflammatory factor participated in biological function of inflammatory response and LXR/RXR signal pathway. Clusterin was firstly identified in EAT and SAT, which is important in the LXR/RXR signaling pathway and may play a protective role in development of CAD. NF-*κ*B p65 as a key protein was engaged in signal pathways including acute phase response and LXR/RXR signal pathway. S100A9 plays vital role in the biological function of inflammatory response, cell-to-cell signaling, and interaction. As shown in Table 3, MMP9 and clusterin expression in EAT of CAD patients was higher than that of non-CAD patients by Western blot analysis. This was in consistence with the results from proteomics. Expression of S100A9 was found to be downregulated in SAT and upregulated in EAT compared to non-CAD patients, and the results were consistent with proteomics data. Proteomics and western blot data for NF-*κ*B in SAT were also in agreement, which was upregulated (Figure 4; Table 3).

## 4. Discussion

Several studies previously explored the differentially expressed proteins between EAT and SAT from CAD patients through the 2DE-MS or MALDI-TOF/TOF methods. However, the numbers of proteins identified from EAT and SAT were small owing to limitations of susceptibility and stability in methodology. Our results identified the largest number of the differentially expressed proteins in EAT and SAT from CAD and non-CAD patients by the iTRAQ-LC/MS/MS method which has higher sensitivity, better sample capacity, higher reproducibility, and more accurate quantitation in comparison with the previous 2-DE technique [16]. By analyzing these differential proteins in EAT and comparing to previous research which found that EAT is involved in lipid metabolism and inflammatory response, our study illustrated more complicated and various biological functions and signal pathways associated with the development of coronary artery atherosclerosis.

### 4.1. Differentially Expressed Proteins from EAT and SAT Are Involved in Biological Processes Associated with Coronary Artery Atherosclerosis

Among the biological processes in which the differentially expressed proteins from EAT and SAT of CAD patients were involved, cell-to-cell signaling and interaction were found to have a great influence on coronary artery atherosclerosis. As shown in Table 2, several adhesion molecules such as ITG, ITGAM, ITGB2, and ITGB3 were found to be highly expressed in EAT of CAD patients compared to that of non-CAD patients. It has been reported that these adhesion molecules regulate and control cell-to-cell and cell-to-matrix interactions [17]. ITGAM and ITGB2 as complement component 3 receptors were involved in inflammatory response. ITGB3 and ITGA2B as platelet glycoprotein IIb of IIb/IIIa which has already been used as therapeutic target to block platelet aggregation in acute coronary syndrome [18, 19]. A similar phenomenon was not found in SAT of CAD patients. These findings suggested that EAT from CAD patients shows more active interaction and adherence between cells and cell-to-matrix and more severe inflammatory response through infiltration of complements and platelets than SAT.

### 4.2. Proteins Differentially Expressed in Both EAT and SAT Are Involved in CAD-Related Metabolic Pathways

#### 4.2.1. Mitochondrial Dysfunction

Mitochondrial dysfunction can result in impaired OXPHOS (Mitochondria mediate oxidative phosphorylation) and increased ROS (reactive oxygen species) generation. These effects facilitate local inflammatory response and vascular atherosclerosis plague formation [20]. In an earlier study, Oana et al. assessed the respiratory function in myocardial fibers of right atrial appendages from patients with coronary heart disease and showed a significant decline of the OXPHOS capacity, electron transport system, and respiratory control ratio for mitochondria energized with complex I substrates. These observations are suggestive of early impairment of complex I-supported respiration in ischemic heart disease [21]. In our present study, for the first time, we found the changes of proteins in mitochondria function in EAT and SAT from CAD patients. In total, 19 proteins differentially expressed in EAT in CAD patients are involved in the mitochondrial dysfunction pathways (Table 2). 8 proteins, namely, SDHA, UQCRC1, NDUFA6, NDUFS4, NDUFA10, NDUFA4, NDUFV1, UQCRB, UQCRC2, and UQCRC1, which are important components of the respiratory chain Complex I/II/III, were dramatically downregulated in EAT of CAD patients, suggesting decreased mitochondrial function. It was speculated that increased ROS production due to mitochondrial dysfunction in EAT of CAD patients may promote inflammation to hasten coronary artery atherosclerosis. Five proteins (ATP5J, NDUFV2, NDUFS6, ATPAF2, and NDUFA8) expressed in SAT of CAD patients were also downregulated, which may suggest that mitochondrial dysfunction and oxidative stress damage are restricted not only to EAT, but also in SAT (Supplementary [Supplementary-material supplementary-material-1]).

#### 4.2.2. LXR/RXR Pathway and Clusterin

Liver X receptors (LXR) of the nuclear receptor superfamily are transcription factors that regulate transcription of several genes involved in cholesterol metabolism which are expressed in intestine, kidney, liver, and adipose tissue. During atherosclerosis, this pathway plays a protective role inhibiting NF-*κ*B pathway to alleviate oxygen stress and restrain proliferation of smooth muscle cells to postpone development of atherosclerosis [22–25]. In this study, there were 19 differentially expressed proteins from EAT of CAD patients involved in this signaling pathway as presented in Table 2. Three proteins including FASN, FDFT1, and PLTP, which control homeostasis of cholesterol metabolism, were found to be downregulated, while S100A8, MMP9, etc., which belong to inflammatory proteins, were found to be upregulated. In referring to the differential proteins expressed in SAT of CAD patients, a total of 10 proteins were found to be involved in LXR/RXR pathway. There has been no research on the expression of the above-mentioned proteins involved in the LXR/RXR pathway in EAT and SAT. Our data suggested that LXR/RXR pathway in SAT is partially activated to inhibit synthesis of cholesterol and accelerate metabolism of cholesterol. In contrast, the activity of this pathway in EAT was decreased, so the homeostasis of cholesterol is damaged, and the inhibitory role of atherosclerosis is also weakened (Supplementary [Supplementary-material supplementary-material-1]).

In the LXR/RXR signaling pathway, for the first time, we found clusterin expression in EAT and SAT to be intimately linked with CAD. Clusterin has been reported to play a role in a wide variety of processes such as lipid transportation, tissue remodeling, and complement inhibition [26, 27]. Clusterin expression in different tissues could exert different roles in coronary artery atherosclerosis. Existence of single cardiomyocytes positive for clusterin after acute myocardial infarction (AMI) suggests a role for clusterin in the protection of cardiomyocytes. Blood plasma levels of clusterin are also elevated in CAD and AMI patients [28]. Clusterin upregulation in distantly located organs should, at least in part, be related to increased ROS levels found in atherosclerotic vessels [29]. In our study, clusterin expression in EAT of CAD patients was also upregulated as per proteomics data, which was further confirmed by western blot method. All these findings, together with our findings, proposed that clusterin expression was activated to protect cardiomyocytes from ischemia and necrosis.

#### 4.2.3. Acute Phase Response and Acute Phase Reaction Protein (APR)

It is well known that inflammatory response is a part of the mechanism of coronary artery atherosclerosis development. In this study, we found that 19 inflammatory proteins differentially expressed in EAT of CAD patients were upregulated. This result was in consistence with recently two studies on transcriptome of EAT. McAninch EA studied the transcriptome of EAT as compared to SAT from patients with coronary artery disease versus valvulopathy and found that EAT is a highly inflammatory tissue enriched with genes involved in endothelial function, coagulation, and immune signaling [30]. Camarena V. et al. evaluated EAT transcriptome in comparison to SAT in coronary artery disease and type 2 diabetes and found that Diabetic EAT was mainly enriched in inflammatory genes which were involved in inflammatory pathways, such as TNF and NF-*κ*B [31]. It is thus inferred that EAT is in the condition of inflammation, where inflammatory cytokines may aggravate atherosclerosis in CAD patients, while SAT of CAD patients is not in an inflammatory state possibly owing to its location far from the heart and owing to its protective role in metabolism (Supplementary [Supplementary-material supplementary-material-1]).

Most of the cytokines taking part in acute phase response are called acute phase reaction proteins (APRs) and can be classified as components of a complement system and coagulation system as well as inflammatory factors. MMP9 belongs to the matrix metalloproteinase (MMP) family, a group of endopeptidases that can degrade ECM proteins to coordinate the vascular remodeling process. Laboratory and clinical data show that MMPs levels and matrix-degrading activity are elevated in vulnerable regions of atherosclerotic plaques. In this study, MMP9 expression in EAT of CAD patients was upregulated by proteomics, which was further confirmed by western blot; thus, this protein is intimately linked with coronary artery atherosclerosis, perhaps by coordinating vascular remodeling and vascular smooth muscle migration. Plasma S100A8/A9 complex has been identified as an early and sensitive biomarker that may discriminate between patients with an acute coronary syndrome and those with stable coronary heart disease [32–34]. There have been several studies on S100A8/S100A9 expression in neutrophils, endothelial cells, smooth muscle cells, and myocardial cells in the initial development of atherosclerosis, but there are few reports on S100A8/S100A9 expression in EAT. In our study, Western blot analysis confirmed that the differential expression of S100A9 was downregulated in SAT and upregulated in EAT of CAD patients, which is consistent with proteomics data.

## 5. Conclusion

Our study reports the largest profiles of differentially expressed proteins in EAT and SAT between CAD patients and non-CAD patients to date. Analysis of function and signaling pathways associated with these proteins suggested that EAT may aggravate the progression of coronary artery atherosclerosis through cell-to-cell signaling and interaction, inflammatory response, dysfunction of lipid metabolism, and mitochondrial dysfunction. In contrast, SAT might play a protective role by improving lipid metabolism and decreasing the inflammatory response. Interestingly, we demonstrated for the first time that the differential proteins were involved in mitochondrial dysfunction and LXR/RXR pathway which both played vital roles in the development of coronary atherosclerosis. These results can serve as the foundation for deeper research on EAT and SAT of CAD patients and may develop further avenues of research for the screening, prevention, and therapy of CAD. The LXR/RXR pathway and these proteins may become therapeutic targets for atherosclerosis. Finally, clusterin, as a component of LXR/RXR pathway and a vital important protective protein, was identified in EAT and SAT, and its mechanism of action and relationship with other signaling pathways or proteins need further investigation.

## 6. Limitation

The sample size of this study was small owing to the rare EAT which is hard to obtain. In order to get two groups of patients whose age, BMI, waist circumference, fasting blood glucose, and lipid profiles were matched, some of patients who did not conform to these terms were excluded.

## Figures and Tables

**Table 1:** Basic information and metabolic index of the CAD and non-CAD groups.

	CAD patients	non-CAD	*P* value
case	6	6	__
Sex (M/F)	4/2	2/4	__
age	59.83±7.57	49.6±8.38	0.053
BMI (kg/m2)	26.30±3.76	25.31±3.24	0.639
WC (cm)	96.83±5.91	89.11±6.30	0.053
FBG (mmol/L)	4.90±0.79	4.70±0.91	0.793
TC (mmol/L)	4.05±1.35	4.6±0.80	0.387
TG (mmol/L)	2.18±0.61	1.83±1.18	0.610
LDL-C (mmol/L)	2.53±1.03	2.92±0.68	0.251
HDL-C (mmol/L)	0.84±0.31	1.04±0.10	0.357
hypertension	6	1	—
Systolic pressure (mmHg)	128.17±14.16	119.22±13.65	0.297
Diastolic pressure (mmHg)	75.17±15.39	80.00±8.02	0.510
diabetes	0	0	—
Statin usage	3	0	—

BMI: body mass index; WC: waist circumference; FBG: fasting blood glucose; TC: cholesterol; TG: triglycerides; LDL-C: low density lipoprotein; HDL-C: high density lipoprotein.

**Table 2:** Changes in differential expression of proteins involved in significant biological functions and signal pathways from EAT and SAT between CAD and non-CAD subjects.

	Differential proteins from EAT (CAD vs non-CAD)	Numbers	Differential proteins from SAT (CAD vs non-CAD)	Numbers
*Biological function*				
Metabolic disorder				
Up	CA1, CA2, CA3, F2, ITGB3, THBS1	6	LPA	1
Down	ATP2A2, FABP4, FABP5, FASN, LIPE, PDE3B	6	APOA2	1
Inflammatory response				
Up	BPI, C3, CAMP, CORO1A, CTSG, CYBB, DEFA1, HCK, IL16, ITGAM, ITGB2, LPA, MMP9, NCKAP1L, ORM1, PROS1, RELA, S100A12, S100A8, SERPINA3, THBS1, THBS4	22	APOL3/APOL4, LPA	2
Down		0	AGT, BPI, HCK, ITGB2, KNG1, S100A12, S100A9, SERPINA1	8
Lipid metabolism				
Up	ALOX5, ATP8A1, CLU, F2, IL16, ITGB3, LTF, MECR, RELA, VTN	10		0
Down	ACOT1, AKR1C1/AKR1C2, AKR1C3, APOA2, FASN, PLTP, SDHA, SUCLA2	8	AGT, ALOX5, KNG1, SOD-1	4
cell-to-cell signaling and interaction				
Up	A2M, C3, C4B, CORO1A, CTSG, ELANE, F10, F2, FCGR3B, FERMT3, FGA, FN1, ICAM3, ITGA2B, ITGAM, ITGB1, ITGB2, LTF, ORM1, RELA, S100A8, SLC4A1, THBS1, ITGB3, VTN, BPI, F5, DEFA1, ASAP2, CAMP, HCK, PROS1, PRTN3	33	FN1	1
Down	PLTP, FERMT2, RRAS2	3	BPI, ITGB1, ITGB2, KNG1, S100A9, BGN, S100A12, AGT	8
free radial Scavenging				
Up	ALOX5, CAMP, CYBB, ELANE, F2, FCGR3B, HBB, HTT, IMMT, ITGAM, ITGB1, ITGB2, ITGB3, LTF, MPO, NGFR, PRTN3, PTPN6	18	FTL	1
Down	CRYAB	1	AGT, ALOX5, HBB, HTT, IMMT, ITGB1, ITGB2, MPOSEPP1, SERPINA1, SOD1	11

*Signal pathway*				
LXR/RXR signal pathway				
Up	FGA, MMP9, CLU, SERPINF2, ORM1, NGFR, C4B, S100A8, RELA, TTR, LPA, C3, VTN	13	FDFT1, LPA	2
Down	APOA2, PCYOX1, FDFT1, FASN, PLTP, HADH	6	APOL1, KNG1, PON1, TF, APOA2, C4B, SERPINA1, AGT	8
Acute phase response				
Up	RELA, ITIH3, TTR, C4BPB, FN1, C3, C1S, SERPINA3, F2, SERPINF2, FGG, SERPI-ND1, ORM1, C4BPA, NGFR, C4B, FGB, FGA, A2M	19	FTL, FN1, FGG	3
Down	APOA2, RRAS2	2	TF, APOA2, C4B, SERPINA1, AGT	5
Mitochondrial dysfunction				
Up	SNCA	1	ATP5J, ATPAF2	2
Down	NDUFA4, SDHA, ATP5J, NDUFV1, ATP5A1, UQCRB, MT-CO2, MAOB, NDUFA6, UQCRC2, NDUFA10, CYB5A, ATPAF2, OGDH, UQCRC1, ATP5F1, M-AOA, NDUFS4	18	NDUFV2, NDUFS6, NDUFA8	3

**Table 3:** Differential comparison of MMP9, NF-*κ*B, clusterin, and S100A9 by proteomics and Western blot methods.

	EAT (CAD vs non-CAD)		

	Proteomics	Western blot	
			*P* value
MMP-9	2.3	2.87	<0.05
Clusterin	2.2	2.9	<0.01
S100A9	1.4	3.2	<0.05

	SAT (CAD vs non-CAD)		

	Proteomics	Western blot	
			*P* value
S100A9	0.3	0.7	<0.05
NF-*κ*B P65	1.2	1.7	>0.05

MMP9: matrix metalloproteinase; NF-*κ*B P65: nuclear factor-kappa B P65 (RelA); S100A9: calgranulin A.

## Data Availability

No data were used to support this study.

## Supplementary Materials

Supplementary Materials Six supplementary materials were provided to help illustrate this study better. Supplementary Table 1 showed detailed information on patients from CAD and non-CAD groups in this study. Supplementary Tables 2 and 3, respectively, showed all of the differentially expressed proteins identified in EAT and SAT between CAD and non-CAD patients in this study. Supplementary Figures 1, 2, and 3, respectively, illustrated the signaling pathways which differentially expressed proteins were involved in, which included mitochondrial dysfunction signaling pathway, LXR/RXR signaling pathway, and acute phase response.
