# Supplementary material for: Comparative Proteome Analysis of Epicardial and Subcutaneous Adipose Tissues from Patients with or without Coronary Artery Disease
Source: Int J Endocrinol. 2019 Aug 25;2019:6976712. doi: 10.1155/2019/6976712 (PMC6732630; doi:10.1155/2019/6976712)
Supplement: Supplementary Materials — Six supplementary materials were provided to help illustrate this study better. Supplementary Table 1 showed detailed information on patients from CAD and non-CAD groups in this study. Supplementary Tables 2 and 3, respectively, showed all of the differentially expressed proteins identified in EAT and SAT between CAD and non-CAD patients in this study. Supplementary Figures 1, 2, and 3, respectively, illustrated the signaling pathways which differentially expressed proteins were involved in, which included mitochondrial dysfunction signaling pathway, LXR/RXR signaling pathway, and acute phase response. [file 6976712.f1.zip › 6976712.f1/Supplementary figure 3.docx]

**Supplementary figure 3. Differentially expressed proteins involved in acute phase response**


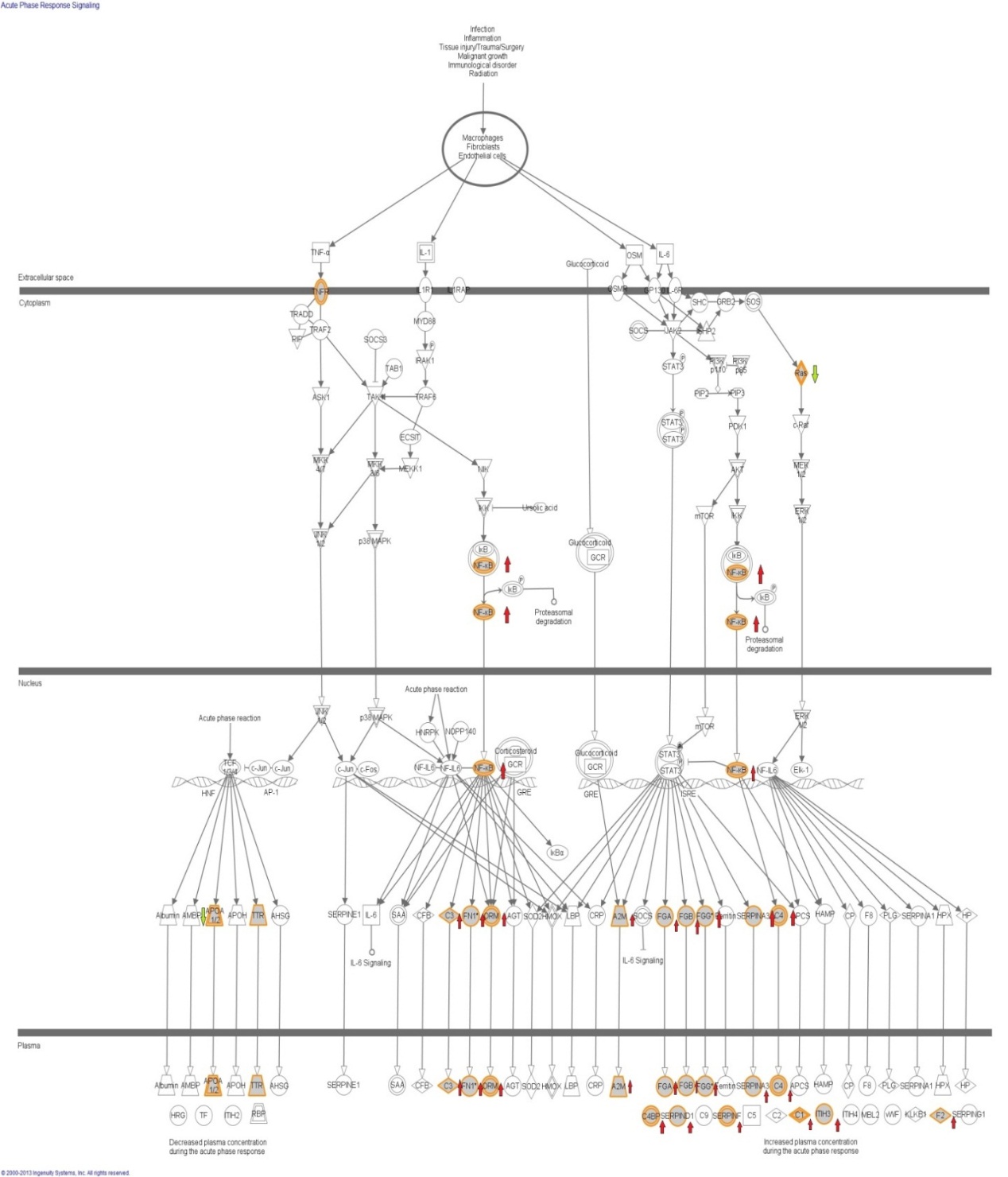


Orange circle indicates differentially expressed proteins, red arrow indicates upregulated proteins, green arrow indicates downregulated proteins.
