# Supplementary material for: Comparative Proteome Analysis of Epicardial and Subcutaneous Adipose Tissues from Patients with or without Coronary Artery Disease
Source: Int J Endocrinol. 2019 Aug 25;2019:6976712. doi: 10.1155/2019/6976712 (PMC6732630; doi:10.1155/2019/6976712)
Supplement: Supplementary Materials — Six supplementary materials were provided to help illustrate this study better. Supplementary Table 1 showed detailed information on patients from CAD and non-CAD groups in this study. Supplementary Tables 2 and 3, respectively, showed all of the differentially expressed proteins identified in EAT and SAT between CAD and non-CAD patients in this study. Supplementary Figures 1, 2, and 3, respectively, illustrated the signaling pathways which differentially expressed proteins were involved in, which included mitochondrial dysfunction signaling pathway, LXR/RXR signaling pathway, and acute phase response. [file 6976712.f1.zip › 6976712.f1/Supplementary table 1.docx]

**Supplementary table 1**. Detailed information on patients from CAD and non-CAD groups.

Detailed information of patients from CAD group

| **Num** | **Age** | **Sex** | **Height**  **(cm)** | **Weight**  **(kg)** | **BMI**  **(Kg/m^2^)** | **Main diagnosis** | **Other diagnosis** |
| --- | --- | --- | --- | --- | --- | --- | --- |
| P1 | 61 | Male | 170 | 67.8 | 23.4 | CAD, STEMI | HTN, HLP, Limb artery occlusion, BPH, digestive tract ulcer |
| P2 | 64 | Male | 174 | 85.2 | 28.1 | CAD, STEMI | HTN, HLP, hyperuricemia |
| P3 | 63 | Male | 165 | 82.2 | 30.2 | CAD | HTN, HLP, gall-stone, gout |
| P4 | 66 | Female | 150 | 46.8 | 20.8 | CAD | subclavian artery steal syndrome, HLP |
| P5 | 45 | Female | 154 | 71 | 29.9 | CAD | HTN, HLP |
| P6 | 60 | Male | 170 | 73 | 25.3 | CAD | HTN, HLP |

Detailed information of patients from non-CAD group

| **Num** | **Age** | **Sex** | **Height**  **(cm)** | **Weight**  **(kg)** | **BMI**  **(Kg/m^2^)** | **Main diagnosis** | **Other diagnosis** |
| --- | --- | --- | --- | --- | --- | --- | --- |
| C1 | 42 | Female | 149 | 61.4 | 27.7 | Valvular heart disease | — |
| C2 | 41 | Female | 169 | 80 | 28.0 | RHD, AF, Pulmonary arterial hypertension | Anemia, Uterine fibroids |
| C3 | 44 | Female | 164 | 61.4 | 22.8 | congenital heart disease, atrial septal defect, Pulmonary arterial hypertension | — |
| C4 | 59 | Male | 170 | 58 | 20.1 | Valvular heart disease | Anemia |
| C5 | 59 | Male | 170 | 74 | 25.6 | RHD, AF, Pulmonary arterial hypertension | HTN, HLP |
| C6 | 53 | Male | 160 | 71 | 27.7 | Valvular heart disease | — |

**Supplementary table**. Detailed information on patients from CAD and non-CAD groups. CAD: coronary heart disease; HTN: hypertension; HLP: hyperlipidemia; RHD: rheumatic heart disease; AF: atrial fibrillation.
