# Supplementary material for: Comparative Proteome Analysis of Epicardial and Subcutaneous Adipose Tissues from Patients with or without Coronary Artery Disease
Source: Int J Endocrinol. 2019 Aug 25;2019:6976712. doi: 10.1155/2019/6976712 (PMC6732630; doi:10.1155/2019/6976712)
Supplement: Supplementary Materials — Six supplementary materials were provided to help illustrate this study better. Supplementary Table 1 showed detailed information on patients from CAD and non-CAD groups in this study. Supplementary Tables 2 and 3, respectively, showed all of the differentially expressed proteins identified in EAT and SAT between CAD and non-CAD patients in this study. Supplementary Figures 1, 2, and 3, respectively, illustrated the signaling pathways which differentially expressed proteins were involved in, which included mitochondrial dysfunction signaling pathway, LXR/RXR signaling pathway, and acute phase response. [file 6976712.f1.zip › 6976712.f1/supplementary table 2.docx]

| **Differentially expressed proteins in EAT between CAD and non-CAD patients** | | | | | | |
| --- | --- | --- | --- | --- | --- | --- |
| **International Protein Index** | **Credibility** | **Times** | **Protein name** | **Entrez Gene Name** | **Location** | **Types** |
| IPI00025344 | 100% | 0.1 | NDUFS6 | NADH dehydrogenase (ubiquinone) Fe-S protein 6, 13kDa (NADH-coenzyme Q reductase) | Cytoplasm | enzyme |
| IPI00027462 | 100% | 0.3 | S100A9 | S100 calcium binding protein A9 | Cytoplasm | other |
| IPI00002335 | 100% | 0.4 | HTT | huntingtin | Cytoplasm | transcription regulator |
| IPI00021854 | 100% | 0.5 | APOA2 | apolipoprotein A-II | Extracellular Space | transporter |
| IPI00218131 | 100% | 0.5 | S100A12 | S100 calcium binding protein A12 | Cytoplasm | other |
| IPI00218916 | 91% | 0.5 | ALOX5 | arachidonate 5-lipoxygenase | Cytoplasm | enzyme |
| IPI00010790 | 100% | 0.6 | BGN | biglycan | Extracellular Space | other |
| IPI00022463 | 100% | 0.6 | TF | transferrin | Extracellular Space | transporter |
| IPI00029061 | 100% | 0.6 | SEPP1 | selenoprotein P, plasma, 1 | Extracellular Space | other |
| IPI00029769 | 100% | 0.6 | HCK | hemopoietic cell kinase | Cytoplasm | kinase |
| IPI00032220 | 100% | 0.6 | AGT | angiotensinogen (serpin peptidase inhibitor, clade A, member 8) | Extracellular Space | growth factor |
| IPI00032258 | 100% | 0.6 | C4B (includes others) | complement component 4B (Chido blood group) | Extracellular Space | other |
| IPI00217561 | 96% | 0.6 | ITGB1 | integrin, beta 1 (fibronectin receptor, beta polypeptide, antigen CD29 includes MDF2, MSK12) | Plasma Membrane | transmembrane receptor |
| IPI00218732 | 100% | 0.6 | PON1 | paraoxonase 1 | Extracellular Space | phosphatase |
| IPI00218733 | 100% | 0.6 | SOD1 | superoxide dismutase 1, soluble | Cytoplasm | enzyme |
| IPI00291328 | 100% | 0.6 | NDUFV2 | NADH dehydrogenase (ubiquinone) flavoprotein 2, 24kDa | Cytoplasm | enzyme |
| IPI00553177 | 100% | 0.6 | SERPINA1 | serpin peptidase inhibitor, clade A (alpha-1 antiproteinase, antitrypsin), member 1 | Extracellular Space | other |
| IPI00796636 | 97% | 0.6 | HBB | hemoglobin, beta | Cytoplasm | transporter |
| IPI00827847 | 100% | 0.6 | BPI | bactericidal/permeability-increasing protein | Plasma Membrane | transporter |
| IPI00007244 | 100% | 0.67 | MPO | myeloperoxidase | Cytoplasm | enzyme |
| IPI00009960 | 100% | 0.67 | IMMT | inner membrane protein, mitochondrial | Cytoplasm | other |
| IPI00186903 | 100% | 0.67 | APOL1 | apolipoprotein L, 1 | Extracellular Space | transporter |
| IPI00215894 | 100% | 0.67 | KNG1 | kininogen 1 | Extracellular Space | other |
| IPI00219034 | 97% | 0.67 | NDUFA8 | NADH dehydrogenase (ubiquinone) 1 alpha subcomplex, 8, 19kDa | Cytoplasm | enzyme |
| IPI00002521 | 100% | 1.5 | ATP5J | ATP synthase, H+ transporting, mitochondrial Fo complex, subunit F6 | Cytoplasm | transporter |
| IPI00922213 | 100% | 1.5 | FN1 | fibronectin 1 | Extracellular Space | enzyme |
| IPI00029168 | 100% | 1.6 | LPA | lipoprotein, Lp(a) | Extracellular Space | other |
| IPI00302796 | 89% | 1.6 | APOL3/APOL4 | apolipoprotein L, 4 | Cytoplasm | transporter |
| IPI01012882 | 96% | 1.6 | FDFT1 | farnesyl-diphosphate farnesyltransferase 1 | Cytoplasm | enzyme |
| IPI00296999 | 100% | 1.7 | ATPA | ATP synthase mitochondrial F1 complex assembly factor 2 | Cytoplasm | other |
| IPI00411626 | 99% | 1.7 | FGG | fibrinogen gamma chain | Extracellular Space | other |
| IPI01014563 | 100% | 2.1 | FTL | ferritin, light polypeptide | Cytoplasm | enzyme |
