# Supplementary material for: Comparative Proteome Analysis of Epicardial and Subcutaneous Adipose Tissues from Patients with or without Coronary Artery Disease
Source: Int J Endocrinol. 2019 Aug 25;2019:6976712. doi: 10.1155/2019/6976712 (PMC6732630; doi:10.1155/2019/6976712)
Supplement: Supplementary Materials — Six supplementary materials were provided to help illustrate this study better. Supplementary Table 1 showed detailed information on patients from CAD and non-CAD groups in this study. Supplementary Tables 2 and 3, respectively, showed all of the differentially expressed proteins identified in EAT and SAT between CAD and non-CAD patients in this study. Supplementary Figures 1, 2, and 3, respectively, illustrated the signaling pathways which differentially expressed proteins were involved in, which included mitochondrial dysfunction signaling pathway, LXR/RXR signaling pathway, and acute phase response. [file 6976712.f1.zip › 6976712.f1/supplementary table 3.doc]

| **Differentially expressed proteins in EAT between CAD and non-CAD patients** | | | | | | |
| --- | --- | --- | --- | --- | --- | --- |
| **International Protein Index** | **Credibility** | **Times** | **Protein name** | **Entrez Gene Name** | **Location** | **Types** |
| IPI00471928 | 100% | 0.4 | ATP5A1 | ATP synthase, H+ transporting, mitochondrial F1 complex, alpha subunit 1, cardiac muscle | Cytoplasm | transporter |
| IPI00177817 | 100% | 0.5 | ATP2A2 | ATPase, Ca++ transporting, cardiac muscle, slow twitch 2 | Cytoplasm | transporter |
| IPI00000856 | 100% | 0.6 | FERMT2 | fermitin family member 2 | Cytoplasm | other |
| IPI00002521 | 100% | 0.6 | ATP5J | ATP synthase, H+ transporting, mitochondrial Fo complex, subunit F6 | Cytoplasm | transporter |
| IPI00005668 | 95% | 0.6 | AKR1C1/AKR1C2 | aldo-keto reductase family 1, member C2 | Cytoplasm | enzyme |
| IPI00011217 | 100% | 0.6 | NDUFS4 | NADH dehydrogenase (ubiquinone) Fe-S protein 4, 18kDa (NADH-coenzyme Q reductase) | Cytoplasm | enzyme |
| IPI00011770 | 100% | 0.6 | NDUFA4 | NADH dehydrogenase (ubiquinone) 1 alpha subcomplex, 4, 9kDa | Cytoplasm | enzyme |
| IPI00017510 | 96% | 0.6 | MT-CO2 | cytochrome c oxidase subunit II | Cytoplasm | enzyme |
| IPI00021369 | 100% | 0.6 | CRYAB | crystallin, alpha B | Nucleus | other |
| IPI00028520 | 100% | 0.6 | NDUFV1 | NADH dehydrogenase (ubiquinone) flavoprotein 1, 51kDa | Cytoplasm | enzyme |
| IPI00029561 | 100% | 0.6 | NDUFA10 | NADH dehydrogenase (ubiquinone) 1 alpha subcomplex, 10, 42kDa | Cytoplasm | transporter |
| IPI00098902 | 100% | 0.6 | OGDH | oxoglutarate (alpha-ketoglutarate) dehydrogenase (lipoamide) | Cytoplasm | enzyme |
| IPI00215746 | 100% | 0.6 | FABP4 | fatty acid binding protein 4, adipocyte | Cytoplasm | transporter |
| IPI00216983 | 100% | 0.6 | CA3 | carbonic anhydrase III, muscle specific | Cytoplasm | enzyme |
| IPI00217232 | 100% | 0.6 | SUCLA2 | succinate-CoA ligase, ADP-forming, beta subunit | Cytoplasm | enzyme |
| IPI00217563 | 100% | 0.6 | ITGB1 | integrin, beta 1 (fibronectin receptor, beta polypeptide, antigen CD29 includes MDF2, MSK12) | Plasma Membrane | transmembrane receptor |
| IPI00220416 | 100% | 0.6 | UQCRB | ubiquinol-cytochrome c reductase binding protein | Cytoplasm | enzyme |
| IPI00291483 | 100% | 0.6 | AKR1C3 | aldo-keto reductase family 1, member C3 | Cytoplasm | enzyme |
| IPI00294398 | 100% | 0.6 | HADH | hydroxyacyl-CoA dehydrogenase | Cytoplasm | enzyme |
| IPI00296999 | 100% | 0.6 | ATPAF2 | ATP synthase mitochondrial F1 complex assembly factor 2 | Cytoplasm | other |
| IPI00305383 | 100% | 0.6 | UQCRC2 | ubiquinol-cytochrome c reductase core protein II | Cytoplasm | enzyme |
| IPI00333838 | 100% | 0.6 | ACOT1 | acyl-CoA thioesterase 1 | Cytoplasm | enzyme |
| IPI00419542 | 100% | 0.6 | LIPE | lipase, hormone-sensitive | Cytoplasm | enzyme |
| IPI00945153 | 97% | 0.6 | NDUFA6 | NADH dehydrogenase (ubiquinone) 1 alpha subcomplex, 6, 14kDa | Cytoplasm | enzyme |
| IPI00964764 | 100% | 0.6 | SDHA | succinate dehydrogenase complex, subunit A, flavoprotein (Fp) | Cytoplasm | enzyme |
| IPI00007797 | 100% | 0.67 | FABP5 | fatty acid binding protein 5 (psoriasis-associated) | Cytoplasm | transporter |
| IPI00008483 | 100% | 0.67 | MAOA | monoamine oxidase A | Cytoplasm | enzyme |
| IPI00012512 | 100% | 0.67 | RRAS2 | related RAS viral (r-ras) oncogene homolog 2 | Plasma Membrane | enzyme |
| IPI00012843 | 100% | 0.67 | PDE3B | phosphodiesterase 3B, cGMP-inhibited | Cytoplasm | enzyme |
| IPI00013847 | 100% | 0.67 | UQCRC1 | ubiquinol-cytochrome c reductase core protein I | Cytoplasm | enzyme |
| IPI00021854 | 100% | 0.67 | APOA2 | apolipoprotein A-II | Extracellular Space | transporter |
| IPI00026781 | 100% | 0.67 | FASN | fatty acid synthase | Cytoplasm | enzyme |
| IPI00029133 | 100% | 0.67 | ATP5F1 | ATP synthase, H+ transporting, mitochondrial Fo complex, subunit B1 | Cytoplasm | transporter |
| IPI00397860 | 100% | 0.67 | CYB5A | cytochrome b5 type A (microsomal) | Cytoplasm | enzyme |
| IPI00025862 | 100% | 1.5 | C4BPB | complement component 4 binding protein, beta | Extracellular Space | other |
| IPI00027436 | 100% | 1.5 | NGFR | nerve growth factor receptor | Plasma Membrane | transmembrane receptor |
| IPI00028064 | 100% | 1.5 | CTSG | cathepsin G | Cytoplasm | peptidase |
| IPI00289966 | 96% | 1.5 | IL16 | interleukin 16 | Extracellular Space | cytokine |
| IPI00298971 | 100% | 1.5 | VTN | vitronectin | Extracellular Space | other |
| IPI00418163 | 100% | 1.5 | C4B (includes others) | complement component 4B (Chido blood group) | Extracellular Space | other |
| IPI00478003 | 100% | 1.5 | A2M | alpha-2-macroglobulin | Extracellular Space | transporter |
| IPI00017696 | 100% | 1.6 | C1S | complement component 1, s subcomponent | Extracellular Space | peptidase |
| IPI00022432 | 100% | 1.6 | TTR | transthyretin | Extracellular Space | transporter |
| IPI00024107 | 100% | 1.6 | SNCA | synuclein, alpha (non A4 component of amyloid precursor) | Cytoplasm | other |
| IPI00029168 | 100% | 1.6 | LPA | lipoprotein, Lp(a) | Extracellular Space | other |
| IPI00029769 | 100% | 1.6 | HCK | hemopoietic cell kinase | Cytoplasm | kinase |
| IPI00216699 | 100% | 1.6 | FERMT3 | fermitin family member 3 | Cytoplasm | enzyme |
| IPI00294004 | 100% | 1.6 | PROS1 | protein S (alpha) | Extracellular Space | other |
| IPI00306159 | 100% | 1.6 | MECR | mitochondrial trans-2-enoyl-CoA reductase | Cytoplasm | enzyme |
| IPI00328550 | 100% | 1.6 | THBS4 | thrombospondin 4 | Extracellular Space | other |
| IPI00847635 | 100% | 1.6 | SERPINA3 | serpin peptidase inhibitor, clade A (alpha-1 antiproteinase, antitrypsin), member 3 | Extracellular Space |  |
| IPI00873416 | 100% | 1.6 | ITIH3 | inter-alpha-trypsin inhibitor heavy chain 3 | Extracellular Space |  |
| IPI00019576 | 100% | 1.7 | F10 | coagulation factor X | Extracellular Space | peptidase |
| IPI00022429 | 100% | 1.7 | ORM1 | orosomucoid 1 | Extracellular Space | other |
| IPI00027769 | 100% | 1.7 | ELANE | elastase, neutrophil expressed | Extracellular Space | peptidase |
| IPI00032402 | 100% | 1.7 | ATP8A1 | ATPase, aminophospholipid transporter (APLT), class I, type 8A, member 1 | Cytoplasm | transporter |
| IPI00218604 | 100% | 1.7 | PTPN6 | protein tyrosine phosphatase, non-receptor type 6 | Cytoplasm | phosphatase |
| IPI00218646 | 100% | 1.7 | CYBB | cytochrome b-245, beta polypeptide | Cytoplasm | enzyme |
| IPI00783987 | 100% | 1.7 | C3 | complement component 3 | Extracellular Space |  |
| IPI00883897 | 98% | 1.7 | RELA | v-rel reticuloendotheliosis viral oncogene homolog A (avian) | Nucleus |  |
| IPI00019568 | 100% | 1.8 | F2 | coagulation factor II (thrombin) | Extracellular Space | peptidase |
| IPI00879573 | 100% | 1.8 | SERPIND1 | serpin peptidase inhibitor, clade D (heparin cofactor), member 1 | Extracellular Space |  |
| IPI00010133 | 100% | 1.9 | CORO1A | coronin, actin binding protein, 1A | Cytoplasm | other |
| IPI00027409 | 99% | 1.9 | PRTN3 | proteinase 3 | Extracellular Space | peptidase |
| IPI00002335 | 100% | 2 | HTT | huntingtin | Cytoplasm | transcription regulator |
| IPI00007244 | 100% | 2 | MPO | myeloperoxidase | Cytoplasm | enzyme |
| IPI00022418 | 100% | 2 | FN1 | fibronectin 1 | Extracellular Space | enzyme |
| IPI00023858 | 100% | 2 | FCGR3B | Fc fragment of IgG, low affinity IIIb, receptor (CD16b) | Plasma Membrane | transmembrane receptor |
| IPI00031620 | 100% | 2 | ICAM3 | intercellular adhesion molecule 3 | Plasma Membrane | transmembrane receptor |
| IPI00292532 | 100% | 2 | CAMP | cathelicidin antimicrobial peptide | Cytoplasm | other |
| IPI00791534 | 100% | 2 | SLC4A1 | solute carrier family 4, anion exchanger, member 1 (erythrocyte membrane protein band 3, Diego blood group) | Plasma Membrane |  |
| IPI00022937 | 100% | 2.1 | F5 | coagulation factor V (proaccelerin, labile factor) | Plasma Membrane | enzyme |
| IPI00218414 | 100% | 2.1 | CA2 | carbonic anhydrase II | Cytoplasm | enzyme |
| IPI00218628 | 100% | 2.1 | ITGA2B | integrin, alpha 2b (platelet glycoprotein IIb of IIb/IIIa complex, antigen CD41) | Plasma Membrane | transmembrane receptor |
| IPI00220350 | 100% | 2.1 | ITGB3 | integrin, beta 3 (platelet glycoprotein IIIa, antigen CD61) | Plasma Membrane | transmembrane receptor |
| IPI00007047 | 100% | 2.2 | S100A8 | S100 calcium binding protein A8 | Cytoplasm | other |
| IPI00021827 | 100% | 2.2 | DEFA1 (includes others) | defensin, alpha 1 | Cytoplasm | other |
| IPI00400826 | 100% | 2.2 | CLU | clusterin | Extracellular Space | other |
| IPI00023407 | 99% | 2.3 | NCKAP1L | NCK-associated protein 1-like | Plasma Membrane | other |
| IPI00027509 | 100% | 2.3 | MMP9 | matrix metallopeptidase 9 (gelatinase B, 92kDa gelatinase, 92kDa type IV collagenase) | Extracellular Space | peptidase |
| IPI00218916 | 91% | 2.3 | ALOX5 | arachidonate 5-lipoxygenase | Cytoplasm | enzyme |
| IPI00217987 | 100% | 2.4 | ITGAM | integrin, alpha M (complement component 3 receptor 3 subunit) | Plasma Membrane | transmembrane receptor |
| IPI00654755 | 100% | 2.4 | HBB | hemoglobin, beta | Cytoplasm |  |
| IPI00827847 | 100% | 2.4 | BPI | bactericidal/permeability-increasing protein | Plasma Membrane |  |
| IPI00021885 | 100% | 2.5 | FGA | fibrinogen alpha chain | Extracellular Space | other |
| IPI00215983 | 100% | 2.5 | CA1 | carbonic anhydrase I | Cytoplasm | enzyme |
| IPI00218131 | 100% | 2.5 | S100A12 | S100 calcium binding protein A12 | Cytoplasm | other |
| IPI00978363 | 100% | 2.6 | ITGB2 | integrin, beta 2 (complement component 3 receptor 3 and 4 subunit) | Plasma Membrane |  |
| IPI00022058 | 100% | 2.7 | ASAP2 | ArfGAP with SH3 domain, ankyrin repeat and PH domain 2 | Nucleus | other |
| IPI00925553 | 100% | 9.9 | IMMT | inner membrane protein, mitochondrial | Cytoplasm |  |
